# Ferulic acid suppresses interleukin‐1β‐induced degeneration of chondrocytes isolated from patients with osteoarthritis through the SIRT1/AMPK/PGC‐1α signaling pathway

**DOI:** 10.1002/iid3.424

**Published:** 2021-06-02

**Authors:** Kewei Du, Xuchen Fang, Ziqiang Li

**Affiliations:** ^1^ Department of Orthopedics Shidong Hospital Affiliated to University of Shanghai for Science and Technology Shanghai China

**Keywords:** chondrocyte degeneration, ferulic acid, interleukin‐1β, osteoarthritis, Sirt1/AMPK/PGC‐1α signaling pathway

## Abstract

**Background:**

Interleukin‐1β (IL‐1β) is involved in osteoarthritis pathogenesis and mediates a series of toxic processes including the production of matrix metalloproteinase and inflammatory regulators which are suppressed by activation of silent information regulator 1 (SIRT1). We aimed to determine the effects of ferulic acid (FA) on IL‐1β‐induced osteoarthritis chondrocyte degeneration.

**Methods:**

We examined the effects of FA on osteoarthritis chondrocyte viability and SIRT1 activation. The impact of FA on IL‐1β‐induced osteoarthritis chondrocyte toxicity was determined by prostaglandin E2 (PGE_2_), nitrite, IL‐6, components of the extracellular matrix, and markers of oxidative stress. Finally, we determined whether these effects were mediated through SIRT1 by inhibiting SIRT1 activity using SIRT1 inhibitor Sirtinol.

**Results:**

We found that FA activated SIRT1/AMPK/PGC‐1α signaling pathway and attenuated IL‐1β‐induced osteoarthritis chondrocyte degeneration by suppressing the production of IL‐6, PGE_2_, nitrite, Collagen I, Runx‐2, MMP‐1, MMP‐3, and MMP‐13, enhancing Collagen II and Aggrecan expression and inhibiting oxidative stress. Inhibition of SIRT1 by Sirtinol attenuated the protective effects of FA.

**Conclusion:**

Our findings reveal that FA prevents IL‐1β‐induced osteoarthritis chondrocyte toxicity, which suggests that FA may be a potential therapy for osteoarthritis and warrants further investigation for its clinical application.

## Graphical abstract

Inhibition of silent information regulator 1 (SIRT1) by Sirtinol attenuated the protective effects of ferulic acid (FA). FA prevents interleukin‐1β (IL‐1β)‐induced osteoarthritis chondrocyte toxicity, which suggests that FA may be a potential therapy.

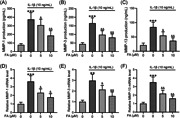

## 1 INTRODUCTION

Osteoarthritis, is a highly prevalent joint disease manifested by synovium inflammation and articular cartilage degeneration, with currently no disease‐modifying medication available.^1^ Interleukin‐1β (IL‐1β), a cytokine produced by chondrocytes, contributes to osteoarthritis pathogenesis.^2^

IL‐1β is detrimental to the normal function of chondrocytes and disrupts extracellular matrix (ECM) integrity. IL‐1β induces the production of multiple proteolytic molecules that promotes the degradation of cartilage.^2^ IL‐1β treatment in chondrocytes promotes matrix metalloproteinase (MMP) production, and upregulates inflammatory regulators, including pro‐inflammatory cytokine IL‐6, prostaglandin E2 (PGE_2_), and nitric oxide, which have been shown to aggravate ECM degradation and cartilage degeneration.^3^, ^4^ Additionally, reactive oxygen species (ROS) and oxidative stress are involved in osteoarthritis progression by causing structural damages to cartilage as well as regulating the inflammatory responses of the synovium.^5^ As a result, IL‐1β treatment of chondrocytes isolated from osteoarthritis patients has been widely used to study osteoarthritis pathogenesis and the development of therapeutic strategies.^6^, ^7^

Silent information regulator 1 (SIRT1) is a histone deacetylase that has been shown to be protective of cartilage by promoting proliferation, differentiation, and survival of chondrocytes and upregulation of genes important for cartilage function such as aggrecan and collagen‐II.^8^, ^9^, ^10^ Several studies have found that the Sirt1/AMPK/PGC‐1α signaling pathway is suppressed in IL‐1β‐stimulated chondrocytes and activation of this pathway in osteoarthritis chondrocytes significantly suppresses inflammatory response, chondrocyte degeneration, and osteoarthritis progression.^6^, ^7^ Thus, therapeutic strategies targeting activation of SIRT1 in osteoarthritis chondrocytes may be effective for the treatment of osteoarthritis. Ferulic acid (FA) is such a candidate. FA activates SIRT1 to suppress osteoporosis^11^ and regulates muscle fiber type formation through this pathway.^12^

FA, a hydroxycinnamic acid present in the walls of plant cells, is a potent antioxidant and has been shown to suppress bone loss by enhancing osteoblast function and inhibiting osteoclast formation. In a previous study, we identified the role of FA in promoting osteogenesis of bone marrow‐derived mesenchymal stem cells.^13^ We hypothesized that FA might suppress IL‐1β‐induced chondrocyte degeneration by activating Sirt1/AMPK/PGC‐1α signaling pathway. In this study, we tested this hypothesis by investigating the potential protective effect of FA against IL‐1β‐induced chondrocyte degeneration and studied the involvement of the Sirt1/AMPK/PGC‐1α signaling pathway in this process.

## 2 MATERIALS AND METHODS

### 2.1 Subjects and tissue collection

Ten patients with primary knee osteoarthritis were enrolled from Shidong Hospital Affiliated to the University of Shanghai for Science and Technology. Osteoarthritis diagnosis was based on the criteria set by the American College of Rheumatology. Among the patients, four were female and six were male, with an average age of 63.8 years. Patients with joint diseases including polyarthritis and rheumatoid, autoimmune disease, developmental dysplasia, skeletal dysplasia, or other post‐septic or posttraumatic arthritis were excluded. Informed consent was acquired from the subjects. This study was approved by the ethics committee of Shidong Hospital Affiliated to the University of Shanghai for Science and Technology (approval number: 2020‐033‐01).

### 2.2 Human primary chondrocyte isolation and culture

Primary chondrocytes from the cartilages separated from the collected tissue samples of enrolled patients were isolated according to a previous study.^6^ Briefly, clean cartilage pieces were dissociated in 0.25% trypsin–EDTA followed by 0.2% collagenase type II for 5 h at 37°C. Chondrocytes were obtained in the pellet following centrifugation for 5 min at 1000 rpm and suspended in DMEM/F12 containing 10% fetal bovine serum and 1% penicillin and streptomycin. The isolated chondrocytes were then maintained at 1 × 10^5^ cells/ml in six‐well plates in a humidified incubator with 5% CO_2_ at 37°C and passaged at a confluence of 80%–90%. Cells were incubated with 10 ng/ml IL‐1β, 10 μM sirtinol, and/or different concentrations of FA (FA and sirtinol were purchased from Sigma‐Aldrich and dissolved in dimethyl sulfoxide. They were added into the culture medium just before the use) for 24 h as indicated. To keep the phenotype consistent, we only used passages 1–2 in this study.

### 2.3 Cell Counting Kit‐8 (CCK‐8) assay

CCK‐8 assay was used to assess chondrocyte viability.^6^ To assess how FA affected chondrocyte viability, cells were maintained in 96‐well plates and were incubated with indicated FA and/or IL‐1β (10 ng/ml) for 24 h. Each well of cells was then treated with 10 μl CCK‐8 at 37°C for 4 h and optical density at 450 nm was obtained.

### 2.4 SIRT1 activity assay

SIRT1 activity was measured by SIRT1 Fluorometric Assay Kit (Sigma‐Aldrich) according to the instruction provided with the kit.

### 2.5 Western blot analysis

Western blot analysis was carried out to assess protein expression according to a previous study.^7^ Briefly, after indicated treatments, cells were homogenized in lysis buffer. Proteins were separated through sodium dodecyl sulfate‐polyacrylamide gel electrophoresis and transferred to polyvinylidene fluoride membrane which was incubated in primary antibodies overnight at 4°C after blocking in 5% fat‐free milk. Proteins were developed with the Enhanced Chemiluminescence Kit following incubation in secondary antibodies (Goat Anti‐Rabbit IgG H&L [HRP], ab205718, 1:2000 or Goat Anti‐Mouse IgG H&L [HRP], ab205719, 1:2000). Anti‐SIRT1 antibody (ab189494, 1:1000), anti‐p‐AMPK antibody (ab133448, 1:1000), anti‐AMPK antibody (ab207442, 1:1000), anti‐PGC‐1α antibody (ab77210, 1:500), and anti‐β‐actin (ab8227, 1:1500) antibody were used. All antibodies were purchased from Abcam.

### 2.6 Quantitative reverse‐transcription polymerase chain reaction (qRT‐PCR)

qRT‐PCR was carried out to measure messenger RNA (mRNA) levels of indicated genes as previously described.^6^ Briefly, complementary DNA was synthesized based on the total RNA extracted from chondrocytes of osteoarthritis patients with a Reverse Transcription Kit (QuantiTect) followed by qRT‐PCR through the CFX96 Real‐Time PCR System. The following genes were analyzed: *SIRT1, Collagen I, Collagen II, Aggrecan, Runx‐2, MMP‐1, MMP‐3, MMP‐13, SOD1*, and *SOD2*. Data were normalized to *GAPDH* mRNA level and analyzed according to the 2^−∆∆Ct^ method. Primer sequences are shown in Table 1.

**Table 1:** Oligonucleotide primer sequences for qRT‐PCR

Gene	Primer direction	Sequence (5ʹ–3ʹ)
SIRT1	Forward	TAGCCTTGTCAGATAAGGAAGGA
	Reverse	ACAGCTTCACAGTCAACTTTGT
Collagen I	Forward	GTGCGATGACGTGATCTGTGA
	Reverse	CGGTGGTTTCTTGGTCGGT
Collagen II	Forward	TGGACGATCAGGCGAAACC
	Reverse	GCTGCGGATGCTCTCAATCT
Aggrecan	Forward	ACTCTGGGTTTTCGTGACTCT
	Reverse	ACACTCAGCGAGTTGTCATGG
Runx‐2	Forward	TCAACGATCTGAGATTTGTGGG
	Reverse	GGGGAGGATTTGTGAAGACGG
MMP‐1	Forward	CTGGCCACAACTGCCAAATG
	Reverse	CTGTCCCTGAACAGCCCAGTACTTA
MMP‐3	Forward	ATTCCATGGAGCAGGCTTTC
	Reverse	CATTTGGGTCAAACTCCAACTGTG
MMP‐13	Forward	TCC CAGGAATTGGTGATAAAGTAGA
	Reverse	CTGGCATGACGCGAACAATA
SOD1	Forward	GGTGAACCAGTTGTGTTGTC
	Reverse	CCGTCCTTTCCAGCAGTC
SOD2	Forward	CAGACCTGCCTTACGACTATGG
	Reverse	CTCGGTGGCGTTGAGATTGTT
GAPDH	Forward	ACAACTTTGGTATCGTGGAAGG
	Reverse	GCCATCACGCCACAGTTTC

Abbreviation: qRT‐PCR, quantitative reverse‐transcription polymerase chain reaction.

### 2.7 Enzyme‐linked immunosorbent assay (ELISA)

Levels of PGE2, IL‐6, MMP‐1, MMP‐3, and MMP‐13 released from chondrocytes were assessed by ELISA by respective ELISA Kits (R&D Systems) as directed by the instructions provided by the manufacturer.

### 2.8 Griess reagent assay

Griess reagent assay was performed to determine nitrite production according to a previous study.^14^ Briefly, the culture medium was incubated with Griess reagent at a volume ratio of 1:1 in dark at room temperature for 10 min and optical density at 540 nm was determined through a microplate reader. Data were normalized to the standard nitrite levels by resuspending sodium nitrite in distilled water.

### 2.9 Assessment of ROS levels

Chondrocyte ROS production following indicated treatments was assessed according to a previous study.^7^ Cells were maintained in 96‐well plates with indicated treatments. After removing the culture medium, chondrocytes were incubated with 10 μM MitoSOX (Invitrogen) for 30 min. The intensity of the fluorescence signal was measured through flow cytometry.

### 2.10 Superoxide dismutase (SOD) activity assay

SOD activity was determined by the SOD Activity Colorimetric Assay Kit (Biovision).

### 2.11 Statistical analysis

Data were analyzed by SPSS and presented as mean ± *SD*. Every experiment has been repeated at least three times. Student's *t* test or one‐way analysis of variance followed by Tukey's post‐hoc test was performed to determine the differences. Differences were considered statistically significant when *p* < .05.

## 3 RESULTS

### 3.1 FA upregulates Sirt1/AMPK/PGC‐1α signaling pathway in chondrocytes from osteoarthritis patients

**Figure 1:**
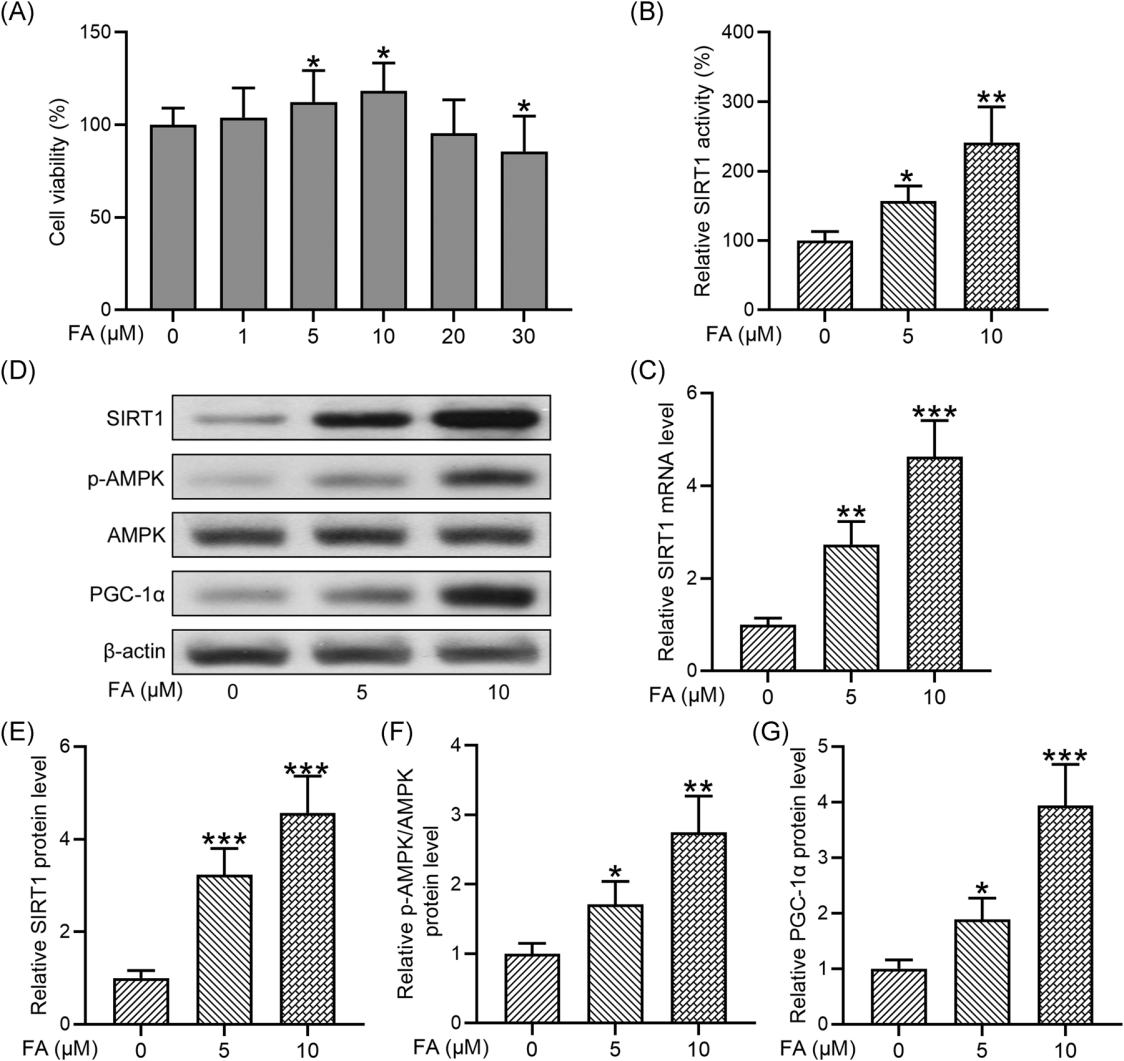
Ferulic acid (FA) increased SIRT1/AMPK/PGC‐1α activation in osteoarthritis chondrocytes. (A) Chondrocyte viability was assessed by Cell Counting Kit‐8 assay. (B) Silent information regulator 1 (SIRT1) activity was assessed by SIRT1 Fluorometric Assay Kit. (C) SIRT1 messenger RNA expression was measured by quantitative reverse‐transcription polymerase chain reaction. (D) Sirt1, p‐AMPK, AMPK, and PGC‐1α protein levels were determined by Western blot analysis. Data were normalized to the control (E–G). *n* = 12 for each group in (A, B). *n* = 4 for each group in (C, E–G). *p < .05, **p < .01, ***p < .001 compared with chondrocytes receiving no stimulation

We first determined whether FA affected the viability of chondrocytes isolated from osteoarthritis patients. CCK‐8 assay showed that FA dose‐dependently affected chondrocyte viability, with concentrations at 5 and 10 μM significantly increasing and 30 μM suppressing the viability (Figure 1A). In the subsequent experiments, we used treated cells with FA at concentrations of 5 and 10 μM.

To determine the impact of FA on the Sirt1/AMPK/PGC‐1α signaling pathway in chondrocytes isolated from osteoarthritis patients, we first examined SIRT1 activity and found that FA dose‐dependently increased SIRT1 activity (Figure 1B). Similarly, FA significantly increased the mRNA level of SIRT1 at 5 and 10 μM (Figure 1C). We then examined protein levels of components of the Sirt1/AMPK/PGC‐1α signaling pathway by Western blot analysis (Figure 1D). Quantification of the Western blot results showed that FA treatments significantly increased SIRT1 protein expression (Figure 1E), AMPK phosphorylation (Figure 1F), and the protein level of PGC‐1α (Figure 1G). These results suggest that FA significantly enhances Sirt1/AMPK/PGC‐1α signaling pathway in chondrocytes isolated from osteoarthritis patients.

### 3.2 FA alleviates IL‐1β‐induced injury to osteoarthritis chondrocytes

**Figure 2:**
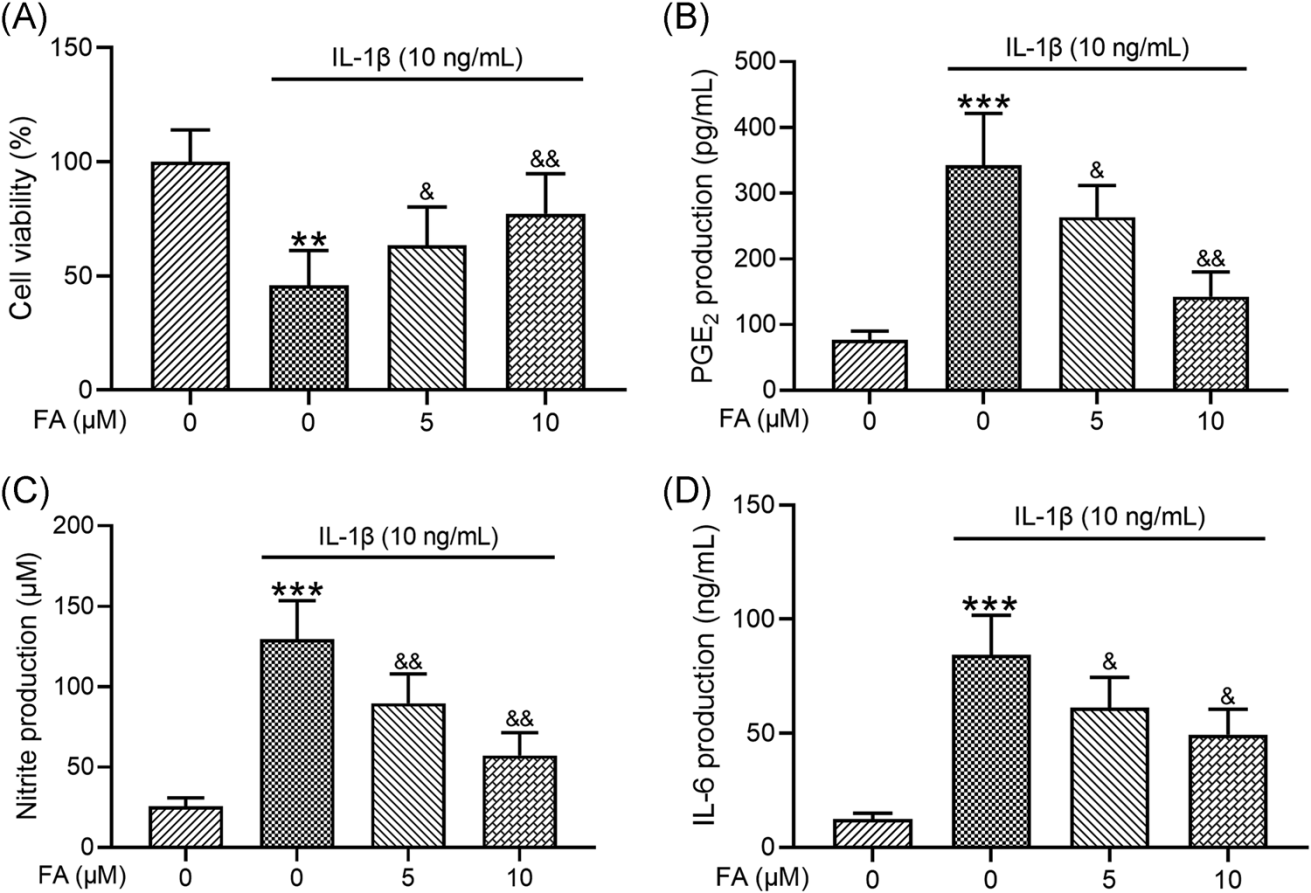
Ferulic acid (FA) alleviates interleukin‐1β (IL‐1β)‐induced osteoarthritis chondrocyte toxicity. (A) Chondrocyte viability was assessed through Cell Counting Kit‐8 assay. (B) PGE2 production was assessed through enzyme‐linked immunosorbent assay (ELISA). (C) Nitrite production was measured by the Griess reagent assay. (D) IL‐6 production was measured by ELISA. *n* = 12 for each group. **p < .01, ***p < .001 compared with chondrocytes receiving no stimulation, ^&^ *p* < .05, ^&&^ *p* < .01 compared with IL‐1β stimulation alone

We next examined how FA affected IL‐1β‐induced cell injury in osteoarthritis chondrocytes. Consistent with previous studies, IL‐1β significantly suppressed the viability of osteoarthritis chondrocytes and co‐treatment of the chondrocytes with FA at 5 and 10 μM dose‐dependently improved cell viability in the presence of IL‐1β (Figure 2A). We then assessed whether FA affected IL‐1β‐induced PGE2, nitrite, and IL‐6 production. ELISA analysis showed that PGE2 production was significantly elevated in osteoarthritis chondrocytes as a result of IL‐1β treatment and co‐treatment of the cells with FA significantly suppressed IL‐1β‐induced PGE2 production (Figure 2B). Examination of nitrite levels showed that IL‐1β significantly increased nitrite production which was suppressed by FA treatment (Figure 2C). Similarly, we found that IL‐1β induced IL‐6 production was suppressed by FA treatment (Figure 2D).

### 3.3 FA alleviates IL‐1β‐induced osteoarthritis chondrocyte degeneration

**Figure 3:**
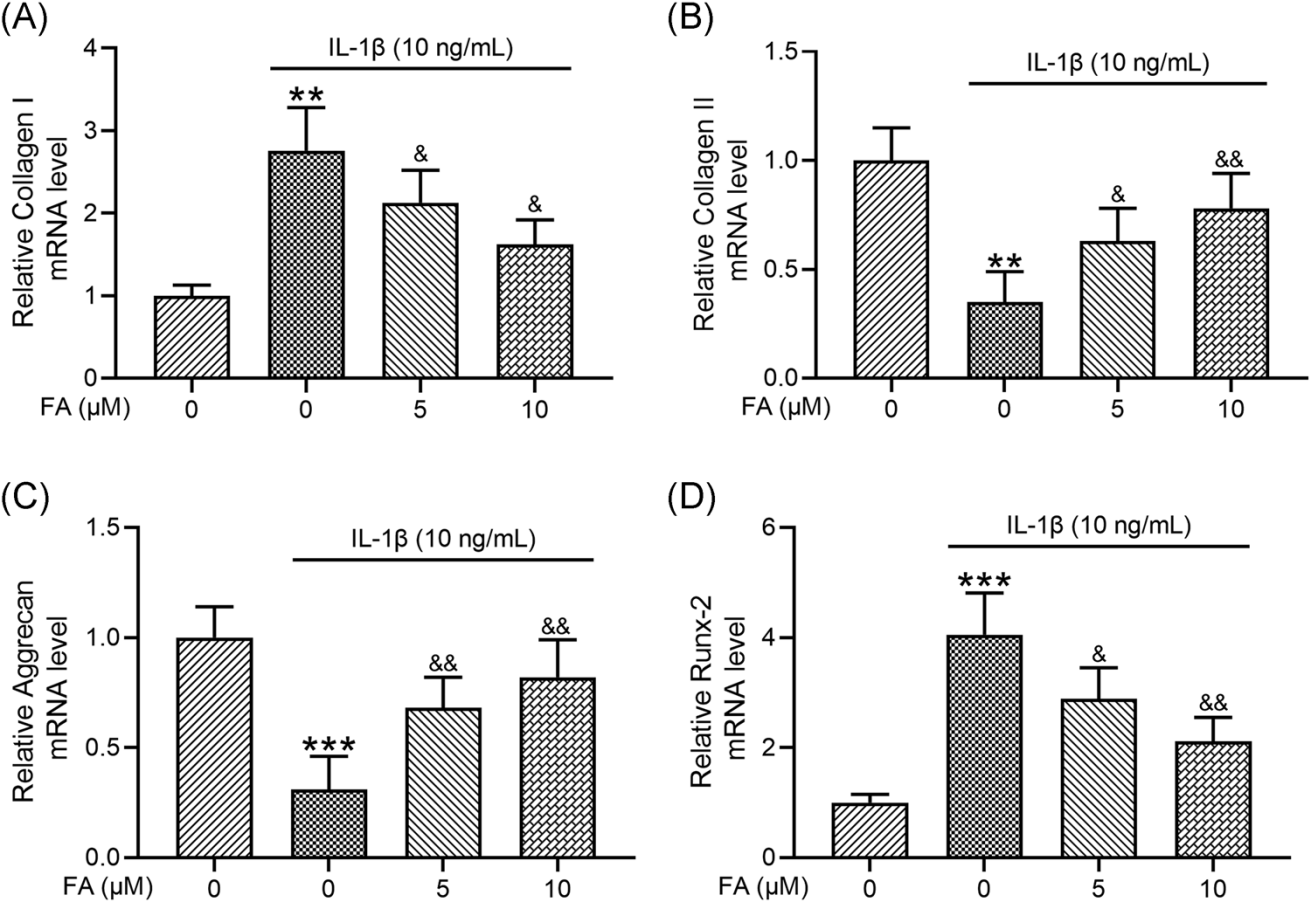
Ferulic acid (FA) alleviates interleukin‐1β (IL‐1β)‐induced osteoarthritis chondrocyte degeneration. Collagen I (A), Collagen II (B), Aggrecan (C), and Runx‐2 (D) messenger RNA (mRNA) levels were determined by quantitative reverse‐transcription polymerase chain reaction. *n* = 4 for each group. **p < .01, ***p < .001 compared with chondrocytes receiving no stimulation, ^&^ *p* < .05, ^&&^ *p* < .01 compared with IL‐1β treatment alone

To investigate how FA impacted IL‐1β‐induced osteoarthritis chondrocyte degeneration, we examined chondrocyte de‐differentiation markers collagen I and Runx‐2 and chondrogenic genes including collagen II and aggrecan. We found that IL‐1β significantly increased collagen I mRNA expression in osteoarthritis chondrocytes which was suppressed by FA treatment (Figure 3A). On the contrary, IL‐1β‐induced suppression of collagen II (Figure 3B), and aggrecan (Figure 3C) mRNA expression was prevented by co‐treatment of the cells with FA. Additionally, IL‐1β‐induced elevation of Runx2 (Figure 3D) mRNA expression was suppressed by FA. These results confirm that FA suppresses IL‐1β‐induced degeneration of osteoarthritis chondrocytes.

### 3.4 FA suppresses IL‐1β‐induced MMP production in osteoarthritis chondrocytes

**Figure 4:**
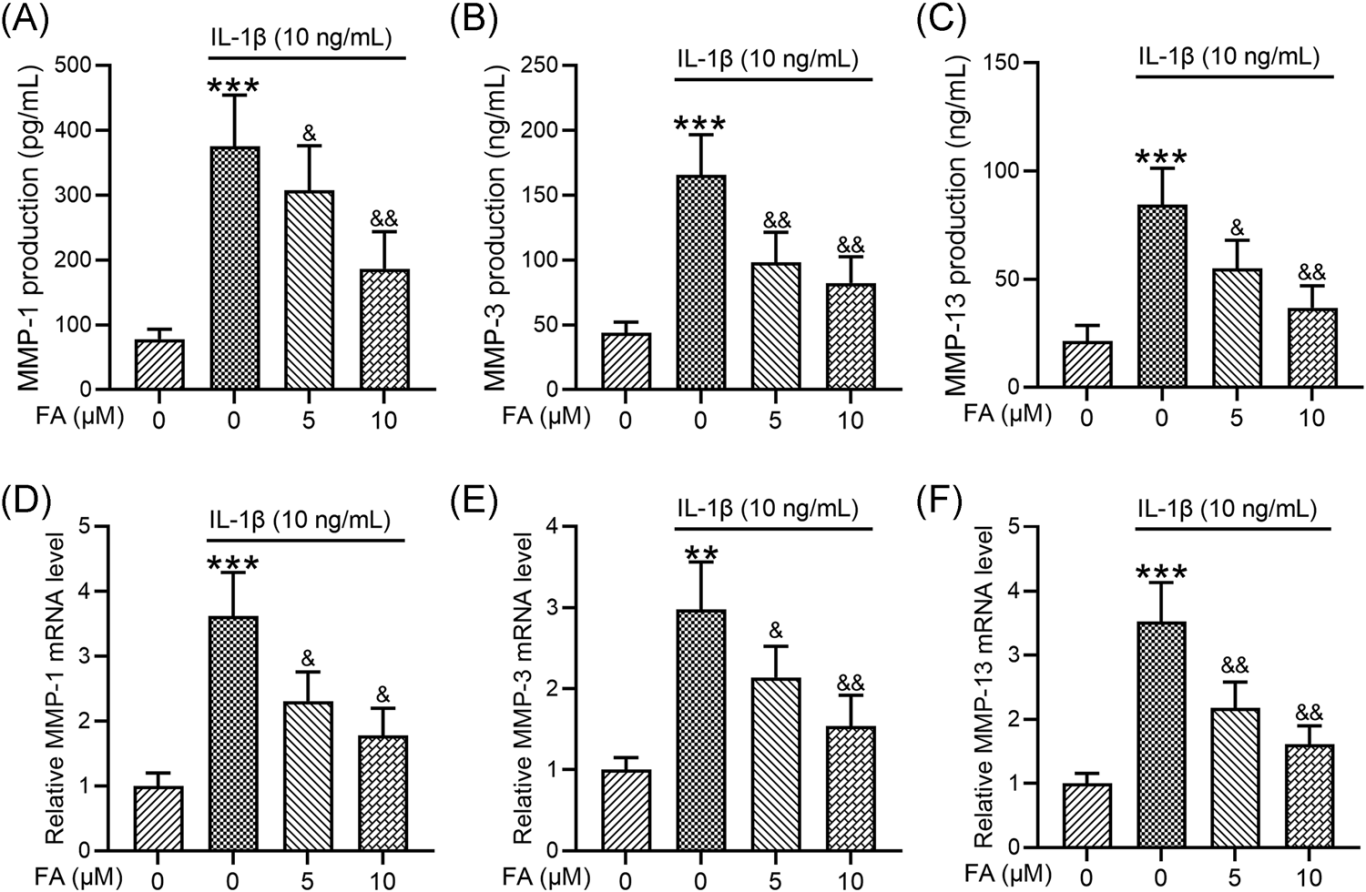
Ferulic acid (FA) alleviates interleukin‐1β (IL‐1β)‐induced matrix metalloproteinase (MMP) production in osteoarthritis chondrocytes. MMP‐1, MMP‐3, and MMP‐13 production were assessed through enzyme‐linked immunosorbent assay (A–C). MMP‐1, MMP‐3, and MMP‐13 messenger RNA (mRNA) expression was measured through quantitative reverse‐transcription polymerase chain reaction (D–F). *n* = 12 for each group in (A–C), *n* = 4 for each group in (D–F). **p < .01, ***p < .001 compared with chondrocytes receiving no stimulation, ^&^ *p* < .05, ^&&^ *p* < .01 compared with IL‐1β stimulation alone

To further investigate how FA suppressed IL‐1β‐induced osteoarthritis chondrocyte degeneration, we determined the levels of MMP production. ELISA analysis showed that IL‐1β significantly elevated MMP‐1 (Figure 4A), MMP‐3 (Figure 4B), and MMP‐13 (Figure 4C) levels and these enhancements were dose‐dependently suppressed by FA treatment. Consistently, we found that MMP‐1 (Figure 4D), MMP‐3 (Figure 4E), and MMP‐13 (Figure 4F) mRNA expression induced by IL‐1β was suppressed by FA treatment.

### 3.5 FA suppresses IL‐1β‐induced oxidative stress in osteoarthritis chondrocytes

**Figure 5:**
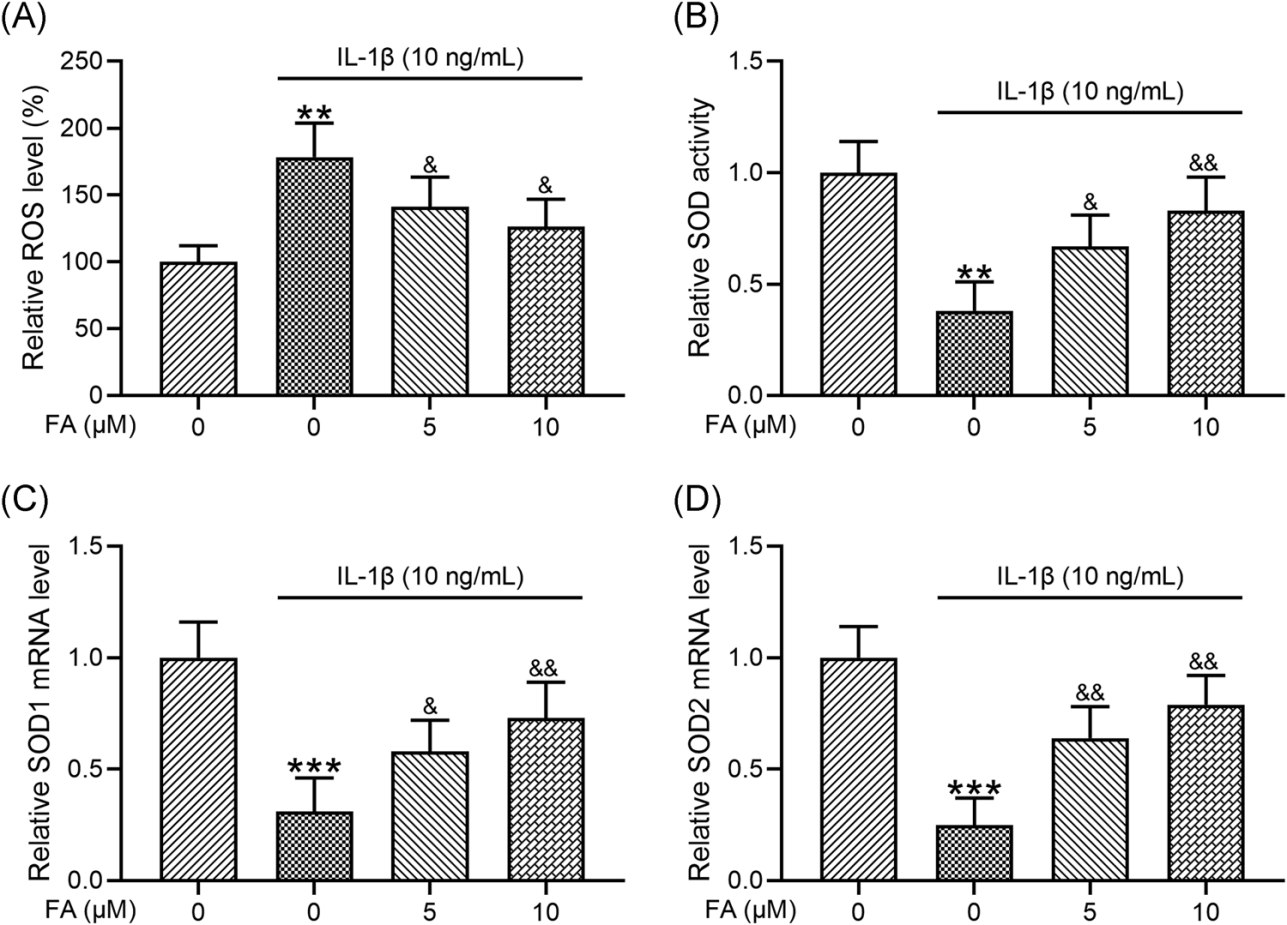
Ferulic acid (FA) alleviates interleukin‐1β (IL‐1β)‐induced oxidative stress in osteoarthritis chondrocytes. (A) Fluorescent intensity of cells was measured to indicate reactive oxygen species (ROS) concentrations. (B) Superoxide dismutase (SOD) activity was examined with a SOD Activity Kit. *n* = 6 for each group. SOD1 and SOD2 messenger RNA (mRNA) levels were tested using quantitative reverse‐transcription polymerase chain reaction (C and D), *n* = 4 for each group. **p < .01, ***p < .001 compared with chondrocytes receiving no stimulation. ^&^ *p* < .05, ^&&^ *p* < .01 compared with IL‐1β stimulation alone

FA is a potent antioxidant. Therefore, we analyzed whether FA could suppress oxidative stress in osteoarthritis chondrocytes stimulated by IL‐1β. Consistent with previous studies, IL‐1β significantly increased ROS levels (Figure 5A), reduced SOD activity (Figure 5B), and decreased the mRNA levels of both SOD1 (Figure 5C) and SOD2 (Figure 5D). Importantly, we found that these changes were suppressed by co‐treatment of the cells with FA, including reduced ROS levels, increased SOD activity, and increased SOD1 and SOD2 mRNA expression.

### 3.6 Inhibition of SIRT1 attenuates the protective effects of FA on IL‐1β‐induced degeneration of osteoarthritis chondrocytes

**Figure 6:**
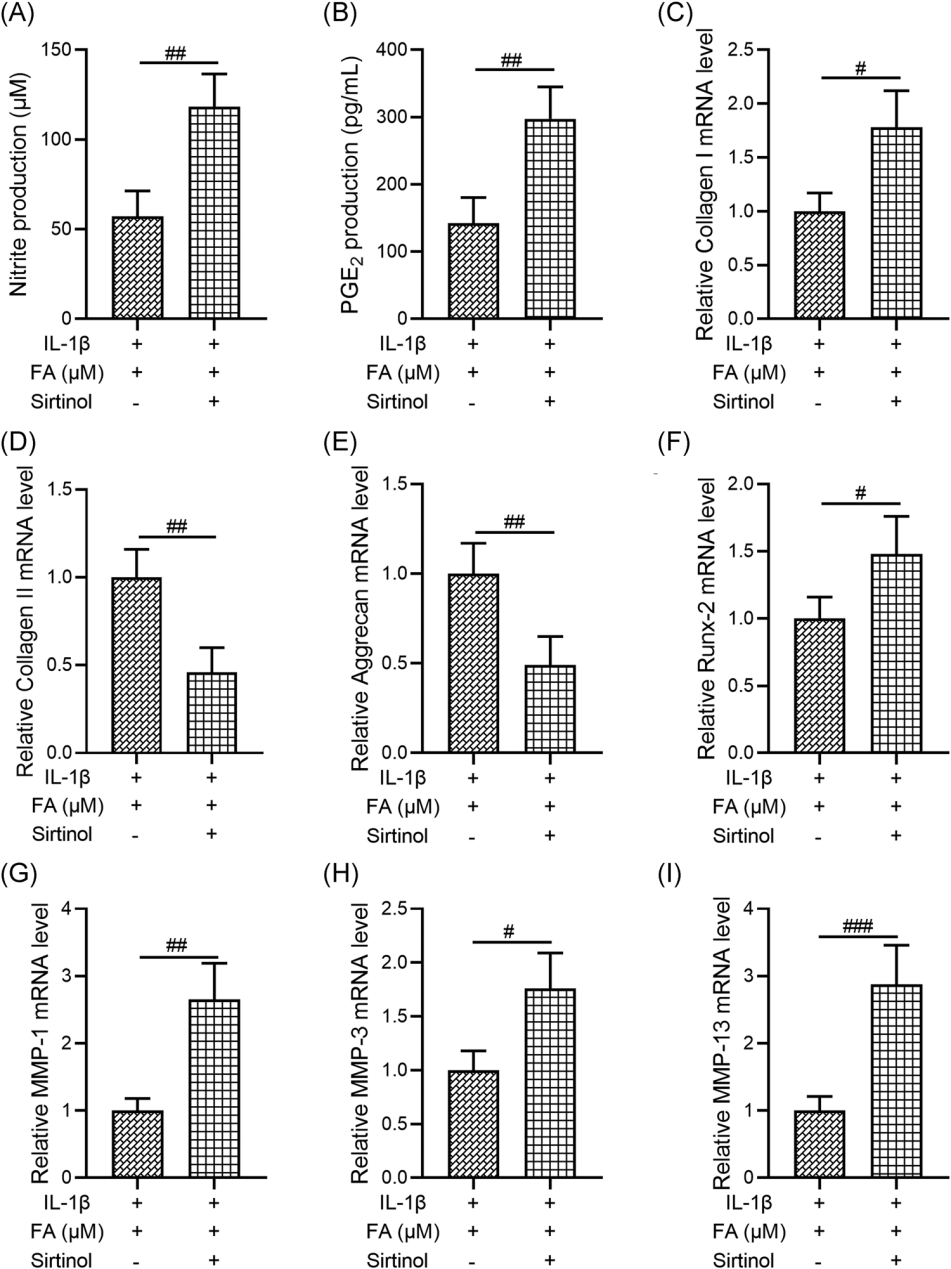
Sirtinol, a silent information regulator 1 (SIRT1) inhibitor, attenuates the protective effects of ferulic acid (FA) on osteoarthritis chondrocyte degeneration caused by interleukin‐1β (IL‐1β). Chondrocytes were co‐treated with 10 ng/ml IL‐1β and 10 μM FA or plus Sirtinol (10 μM) for 24 h. (A) Nitrite production was determined by the Griess reagent assay. (B) PGE2 production was measured by enzyme‐linked immunosorbent assay. *n* = 12 for each group. Collagen I (C), Collagen II (D), Aggrecan (E), Runx‐2 (F), MMP‐1 (G), MMP‐3 (H), and MMP‐13 (I) messenger RNA (mRNA) expression was measured by quantitative reverse‐transcription polymerase chain reaction. *n* = 4 in each group. ^#^ *p* < .05, ^##^ *p* < .01, ^###^ *p* < .001

Finally, as FA significantly enhanced the Sirt1/AMPK/PGC‐1α signaling pathway, we investigated whether suppression of SIRT1 activity by Sirtinol, a SIRT1 inhibitor, could abolish the protective effects of FA on IL‐1β‐induced degeneration of osteoarthritis chondrocytes. We found that, compared to cells treated with both IL‐1β and FA, osteoarthritis chondrocytes treated with IL‐1β, FA, and Sirtinol showed increased nitrite production (Figure 6A), increased PGE_2_ production (Figure 6B), increased mRNA levels of Collagen I (Figure 6C) and Runx‐2 (Figure 6F), decreased mRNA levels of Collagen II (Figure 6D) and Aggrecan (Figure 6E), and promoted MMP‐1 (Figure 6G), MMP‐3 (Figure 6H), and MMP‐13 (Figure 6I) mRNA expression. The differences between the two groups were all statistically significant. These results suggest that inhibition of SIRT1 activity significantly suppressed the protective effects of FA against IL‐1β‐induced osteoarthritis chondrocyte degeneration.

## 4 DISCUSSION

This study investigated the potential of FA in suppressing IL‐1β‐induced degeneration of osteoarthritis chondrocytes and explored the molecular mechanism underlying this regulation. We showed that 5 and 10 μM significantly increased the viability of chondrocytes isolated from osteoarthritis patients and activated Sirt1/AMPK/PGC‐1α signaling pathway. Our results indicate that FA effectively prevented IL‐1β‐induced toxicity in osteoarthritis chondrocytes. FA rescued chondrocyte viability, and suppressed IL‐1β‐induced production of PGE2, nitrite, and IL‐6. FA prevented IL‐1β‐induced chondrocyte degeneration by suppressing the expression of chondrocyte de‐differentiation markers collagen I and Runx‐2 and restoring the expression of chondrogenic genes including collagen II and aggrecan as well as suppressing the production of MMP proteins. Additionally, our study also showed that FA effectively suppressed IL‐1β‐induced oxidative stress. Our findings suggest that SIRT1 activity is involved in mediating the protective effects of FA against IL‐1β‐induced toxicity and inhibition of SIRT1 by Sirtinol significantly attenuated the suppressive effects on IL‐1β‐induced osteoarthritis chondrocyte degeneration. Our study for the first time identified a link between FA‐mediated osteoarthritis chondrocyte protection and SIRT1/AMPK/PGC‐1α signaling pathway and first showed involvement of reduced oxidative stress in this regulation.

Chondrocytes are a key component of the articular cartilage that control cartilage function and structure through regulation of the ECM, including production and turnover of MMPs and collagens.^15^ Increased production of MMPs leads to the breakdown of cartilage matrix. Additionally, chondrocytes also secret pro‐inflammatory cytokines including IL‐6 and other inflammatory regulators during osteoarthritis progression resulting in disrupted homeostasis of the cartilage.^16^ In this study, we took advantage of IL‐1β‐induced osteoarthritis chondrocyte degeneration to model osteoarthritis progression in vitro and confirmed that treatment of osteoarthritis chondrocytes leads to reduced chondrocyte viability, increased production of ECM degradation markers, and inflammatory mediators and increased oxidative stress.

FA is a naturally occurring polyphenol that possesses many physiological functions such as antioxidant and anti‐inflammatory effects and a protective role against bone loss. We showed that FA dose‐dependently affected the survival of human osteoarthritis chondrocytes with lower concentrations (5 and 10 μM) promoting survival and higher concentration (30 μM) slightly reducing the viability of the cells. We tested both of the lower concentrations in IL‐1β‐treated osteoarthritis chondrocytes and found FA dose‐dependently suppressed IL‐1β‐induced osteoarthritis chondrocyte degeneration.

Inflammatory mediators including PGE2 and nitric oxide are involved in osteoarthritis pathogenesis and are generated in the cartilage of osteoarthritis patients.^17^ Excessive PGE2 and nitric oxide disrupt ECM metabolism and promote its destruction. Our results revealed that co‐treatment of osteoarthritis chondrocytes with FA and IL‐1β suppressed IL‐1β‐induced production of IL‐6 as well as these inflammatory mediators, indicating dampening of the inflammatory response of the cells. In fact, FA has been shown to have an anti‐inflammatory effect on multiple other disease settings such as respiratory distress syndrome induced by lipopolysaccharide and hepatotoxicity induced by formaldehyde.^18^, ^19^ Consistent with our observation, other studies have also reported an anti‐inflammatory role of FA in maintaining bone homeostasis, such as the anti‐inflammatory effect of FA in collagen‐induced arthritis.^20^ Our study together with others suggest nutritional supplementation of this nutraceutical may exert a potential therapeutic on osteoarthritis by suppressing inflammation.^21^

Under conditions of inflammation, chondrocytes actively change and remodel the structure and composition of ECM which compromises the quality of ECM, leading to cartilage degeneration.^22^ During osteoarthritis progression, production of ECM components is substantially altered, including collagen I, collagen II, aggrecan, and Runx‐2, which changes the microenvironment of the cartilage and disrupts its integrity.^22^ Additionally, MMPs are also shown to be overexpressed in osteoarthritis patients and exert major roles in cartilage degradation.^23^ In fact, a recent study has identified a significant elevation of serum MMP‐3 level in patients with knee osteoarthritis and indicates that this level may serve as a prognostic marker for cartilage destruction.^24^ Inhibition of MMP expression is a potential treatment for osteoarthritis.^23^ Importantly, our study revealed that FA suppressed MMP‐1, MMP‐3, and MMP‐13 production stimulated by IL‐1β in vitro, suggesting that FA may be effective for restoring ECM composition and structure to halt osteoarthritis progression.

While many factors contribute to cartilage degeneration in osteoarthritis patients, increased oxidative stress is closely associated with osteoarthritis progression.^5^ Patients with osteoarthritis have been found to have high levels of ROS and oxidative stress.^5^ FA is a potent antioxidant that has been shown to suppress oxidative stress in various disease models.^25^ Importantly, our study showed significantly reduced production of oxidative markers when osteoarthritis chondrocytes were incubated with both FA and IL‐1β.

In searching for the potential molecular mechanism underlying FA‐mediated suppression of IL‐1β‐induced osteoarthritis chondrocyte degeneration, we identified the SIRT1/AMPK/PGC‐1α signaling pathway might be an important cascade mediating the protective effects of FA. A previous study has shown that FA activates SIRT1 to protect against osteoporosis in a rat model.^11^ Additionally, another study also indicated that SIRT1 signaling activation is involved in the antioxidant response of mechanical stretch in human mesenchymal stem cells.^26^ We, therefore, investigated the potential link between FA and the SIRT1/AMPK/PGC‐1α signaling pathway in OC chondrocyte protection, and our study showed that FA treatment leads to enhanced activation of SIRT1/AMPK/PGC‐1α signaling pathway. Consistently, we also found that inhibition of SIRT1 attenuated FA‐mediated protective effects against IL‐1β‐induced toxicity to osteoarthritis chondrocyte.

One limitation of this study is that we only investigated the effect of FA on IL‐1β‐induced toxicity in osteoarthritis chondrocytes but did not isolate chondrocytes from healthy individuals or patients in the early stage of osteoarthritis. One future direction of this study will be to explore the protective effects of FA on chondrocytes isolated from patients with less severe osteoarthritis. Additionally, because Sirt1 is a histone deacetylase involved in the regulation of various gene expressions, it is possible that other players may be involved in the regulation of chondrocyte protection and antioxidant response of FA. One future direction of this study is, therefore, to dissect out more detailed molecular mechanism and screen for gene expression profiles, to provide a better picture when exploring FA as a therapeutic strategy for osteoarthritis.

In summary, our study suggests that FA possesses powerful protective effects on IL‐1β‐induced osteoarthritis degeneration by preventing ECM degradation, suppressing inflammation responses, and inhibiting oxidative stress through regulation of the Sirt1/AMPK/PGC‐1α signaling pathway. These findings further confirm that FA may be a potential therapy for osteoarthritis and warrant further investigation on its clinical application.

## CONFLICT OF INTERESTS

The authors declare that there are no conflict of interests.
